# Angiogenesis Analyzer for ImageJ — A comparative morphometric analysis of “Endothelial Tube Formation Assay” and “Fibrin Bead Assay”

**DOI:** 10.1038/s41598-020-67289-8

**Published:** 2020-07-14

**Authors:** Gilles Carpentier, Sarah Berndt, Ségolène Ferratge, Wayne Rasband, Muriel Cuendet, Georges Uzan, Patricia Albanese

**Affiliations:** 1 Gly-CRRET Research Unit 4397, Université Paris-Est Créteil, 61 avenue du général de Gaulle, 94010 Créteil, France; 2 School of Pharmaceutical Sciences, Université de Genève, Rue Michel Servet 1, 1211, Geneva, 4 Switzerland; 3 INSERM U 1197, Hôpital Paul Brousse, Villejuif, France; 4 U. S. National Institutes of Health, Bethesda, Maryland USA

**Keywords:** Software, Phase-contrast microscopy, Angiogenesis

## Abstract

Angiogenesis assays based on *in vitro* capillary-like growth of endothelial cells (EC) are widely used, either to evaluate the effect of anti- and pro-angiogenesis drugs of interest, or to test and compare the functional capacities of various types of EC and progenitor cells. Among the different methods applied to study angiogenesis, the most commonly used is the “Endothelial Tube Formation Assay” (ETFA). In suitable culture conditions, EC form two-dimensional (2D) branched structures that can lead to a meshed pseudo-capillary network. An alternative approach to ETFA is the “Fibrin Bead Assay” (FBA), based on the use of Cytodex 3 microspheres, which promote the growth of 3D capillary-like patterns from coated EC, suitable for high throughput *in vitro* angiogenesis studies. The analytical evaluation of these two widely used assays still remains challenging in terms of observation method and image analysis. We previously developed the “Angiogenesis Analyzer” for ImageJ (AA), a tool allowing analysis of ETFA-derived images, according to characteristics of the pseudo-capillary networks. In this work, we developed and implemented a new algorithm for AA able to recognize microspheres and to analyze the attached capillary-like structures from the FBA model. Such a method is presented for the first time in fully automated mode and using non-destructive image acquisition. We detailed these two algorithms and used the new AA version to compare both methods (*i.e*. ETFA and FBA) in their efficiency, accuracy and statistical relevance to model angiogenesis patterns of Human Umbilical Vein EC (HUVEC). Although the two methods do not assess the same biological step, our data suggest that they display specific and complementary information on the angiogenesis processes analysis.

## Introduction

Angiogenesis, the growth of new blood vessels from pre-existing ones, is a complex and critical process that takes place during vertebrate development, in specific physiological conditions in adult individuals and during different pathologies^1,2^. Endothelial cells (EC) represent the main cell type engaged in this process. Under appropriate stimuli, these cells sprout from a root vessel, migrate, proliferate, then align and ultimately form tubes^2,3^. Accurate and objective evaluation of these phenomena is necessary for the full comprehension of this process, for both fundamental approaches and pharmacological applications. For example, the use of anti-angiogenic molecules still represents a promising therapeutic strategy to impede the development of solid tumors^4,5^. Other fields of interest, such as stem cell research, also require the evaluation of angiogenic capacities of EC progenitors as well as quantitative tools to monitor their proliferation and differentiation capacities^3,6^. In this context, *in vitro* as well as *in vivo* experimental models have been developed to evaluate angiogenesis features, to screen a variety of new angiostatic molecules and to study their properties^7,8^. Most *in vitro* angiogenesis models were designed based on the so-called “sprouting angiogenesis” differentiation process, whereby pseudo-capillary formation mimics several steps of the *de novo* angiogenesis. The “Endothelial Tube Formation Assays” (ETFA), based on the original design by Montesano and coll^9,10^. is now extensively used and adapted to various models for the screening and study of pro- or anti-angiogenic compounds^11,12^.

An alternative *in vitro* model, named the Fibrin Bead Assay (FBA) is now widely used since its initial description^13^. This test uses a culture of EC at the surface of ∼200 µm-sized Cytodex-3 microspheres embedded in a 3D fibrin matrix, in the presence of normal human dermal fibroblast (NHDF) as feeder cells. These stromal cells provide a perfect mix of various angiogenic growth factors: hepatocyte growth factor (HGF), transforming growth factor alpha (TGF-α), angiopoietin-1 (Ang-1), as well as matrix molecules, matrix-modifying proteins and matricellular proteins (e.g. procollagen C endopeptidase enhancer 1, secreted protein acidic and cysteine-rich (SPARC), transforming growth factor-β-induced protein Ig-H3 (βIgH3) and insulin-like growth factor binding protein 7 (IGFBP7))^12^.

The adaptation of the FBA model into 96-well plates allows high-throughput drug screening using small amounts of the tested products^12,14–17^. This method has the advantage of being closely related, in terms of 3D sprouting freedom with a solid anchorage point, to an *in vivo* assay known as the “Rat Aortic Sprouting Assay”. In addition, the FBA test avoids the use of animal experimentation and favors good repeatability^12^.

Although the qualitative features of both ETFA and FBA have been well described, better quantification methods are needed. Indeed, these assays still present technical flaws, due to physical, optical and computer constraints^12^. Among technical difficulties lays the focus default due to meniscus formed by culture gels, especially in small culture wells used for high-throughput microscopy in FBA experiments. Another difficulty is the choice of the most appropriate acquisition method (fluorescence or phase contrast microscopy) and associated software tool for image analysis. Finally, special consideration must be given to the morphologic parameters to be taken into account for optimal statistical analysis.

Here, it is proposed to compare ETFA and FBA models by using a customized version of the previously proposed “Angiogenesis Analyzer”^18^, a simple and precise tool built in the ImageJ environment^19^ and formerly used to quantify the ETFA experiment images. The “Angiogenesis Analyzer” program was conceived to extract characteristic points and elements of endothelial cells network. This image analysis software was successfully used to characterize meshed and/or branched structures in more than 150 different studies (http://image.bio.methods.free.fr/ImageJ/?Angiogenesis-Analyzer-for-ImageJ&artpage=6-6#outil_sommaire_6), which include endothelial *in vitro* ETFA cell differentiation in phase contrast^20,21^, fluorescence microscopy^22^, and *in vivo* studies using the mouse retina angiogenesis model^23,24^. The “Angiogenesis Analyzer” has also been used to analyze branching in alternative meshed or branched biological scaffolds unrelated to angiogenesis, as reported for the characterization of diatom silicate structures in phytoplankton research^25^, or for *in vitro* neuritic branching analysis in cultured neurons^26^.

The “Angiogenesis Analyzer” software works as an integrated program, avoiding the requirement for image pretreatment using additional plugins, or online computation services on distant servers, such as required by other available solutions^27^. The “Angiogenesis Analyzer” is free and open source, easy to customize and compatible with several OS platforms (Linux, MacOS and Windows) thanks to a Java execution environment. Moreover, it can be used to obtain basic hierarchical data in the form of series of vectorial objects by modeling the analyzed structure at different levels, while most other solutions afford basic measurements as total tree lengths and meshes areas under the form of binary measurements^28^.

We previously separately validated “Angiogenesis Analyzer” for both ETFA^29^ and FBA models^14,15,30^. Here, using the customized “Angiogenesis Analyzer”, we compared the reliability, accuracy and statistical relevance of parameters obtained from two sets of FBA and ETFA experiments, using both activating and inhibiting conditions of angiogenesis on HUVEC cells. These cells spontaneously differentiated, aligned and branched in the FBA model, and formed an irregular meshed network in the ETFA experiments. Cellular organization images were acquired through phase contrast microscopy and analyzed with their corresponding algorithms that were described in details for both model. The FBA algorithm described in this work is the first solution available for FBA fully automated computer analysis using phase contrast microscopy, allowing thousands of microbead analysis per day on a personal computer, without user intervention. By comparative analysis of measurements we observed that sprouting initiation capacities in FBA seem related to meshing development in ETFA, while length of pseudo-vascular tree in FBA is rather associated to a later stage of development. Our data suggest that the two methods do not assess the same biological step and display specific and complementary information in the analysis of angiogenesis processes.

## Results

### FBA image analysis

**Figure 1:**
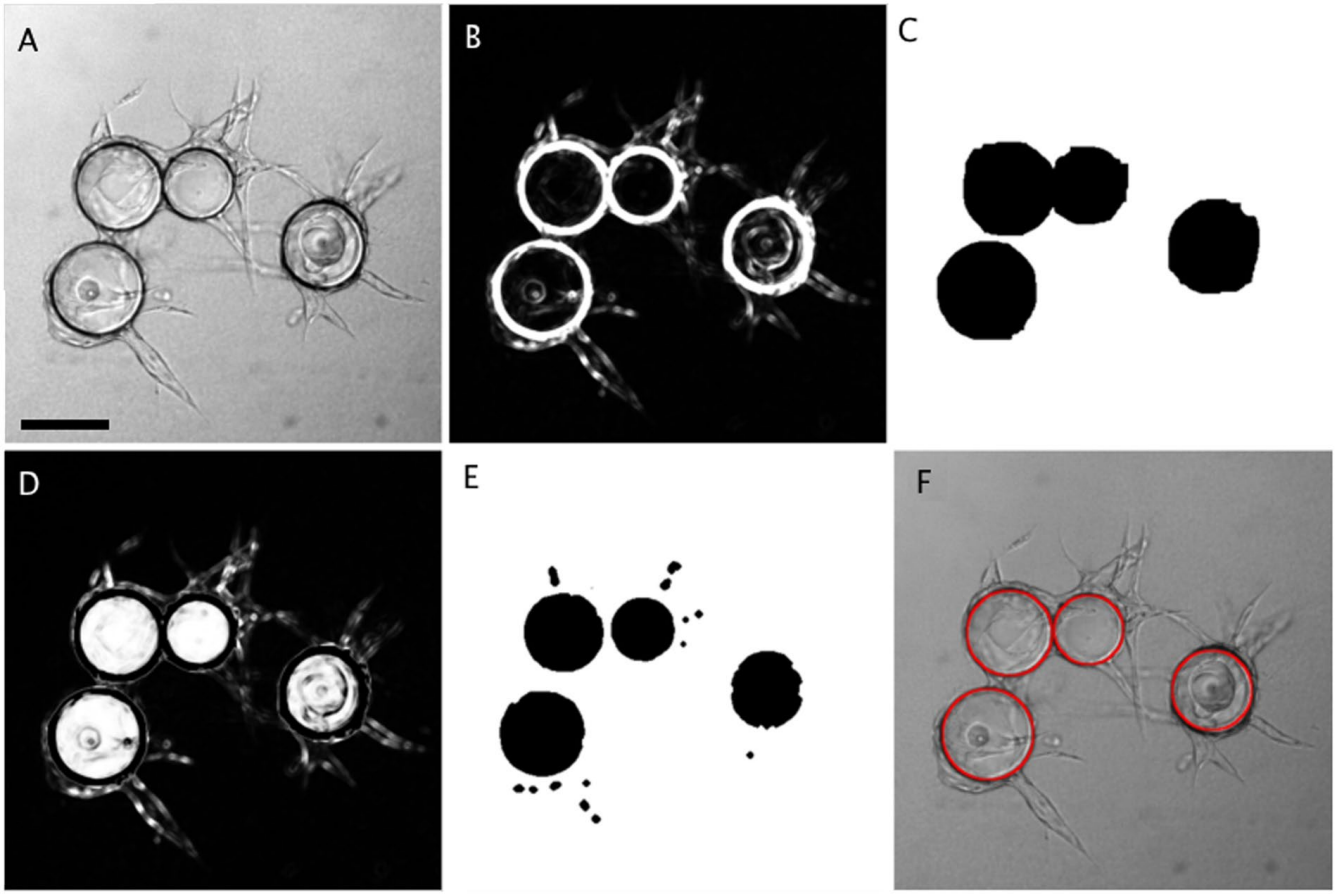
Sphere detection in FBA. (**A**) initial image observed in phase contrast. (**B**) edge-enhanced image (§1-2). (**C**) first binary mask of spheres (§3). (**D**) difference between B and C, highlighting sphere interiors (§4). (**E**) binary mask delineating the interior of the spheres (§4). (**F**) final detection of the sphere edges (red) (§5). “§” labels refer to the different steps described in the Results section. Scale bar: 200 µm.

**Figure 2:**
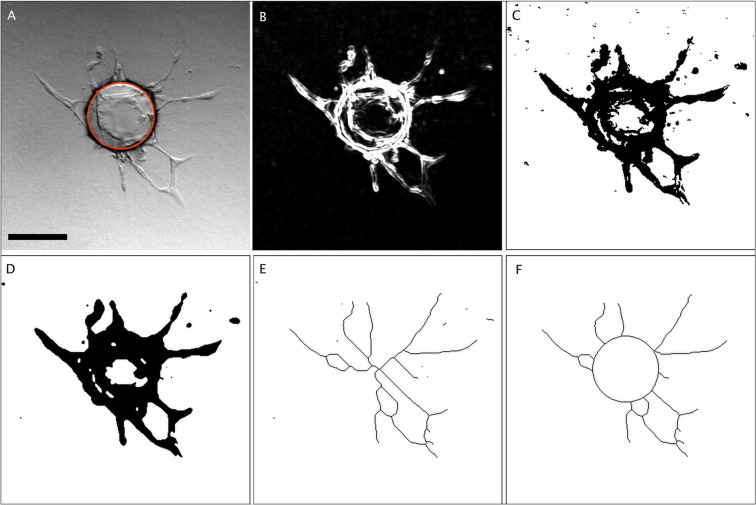
Tree detection in FBA. (**A**) initial sample image exhibiting the detected sphere as a red vector overlay (§1-§5). (**B**) enhancement of high gradients and background removal (§6-§7). (**C**) “Mean” threshold. (**D**) final binary segmentation (§8). (**E**) skeleton of the binary segmentation (§9). (**F**) final skeleton after clearing of the sphere interior (§9). “§” labels correspond to steps described in the Results section. Scale bar: 200 µm.

**Figure 3:**
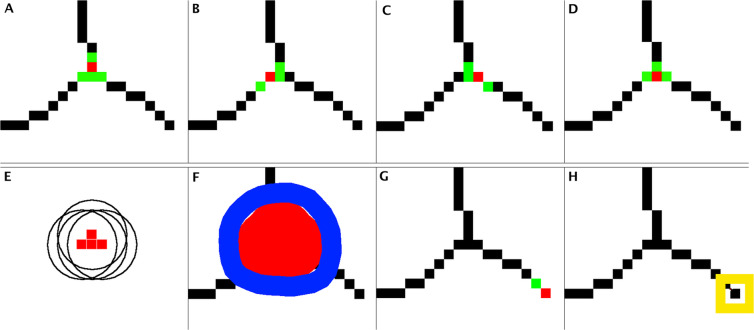
Detection of *Junctions* and *Extremities* in skeletonized trees obtained through different steps. (**A to D**) Represent four pixels (in red) each linked to three neighborning pixels (green). (**E**) The junction between the three binary segments is composed of the four nodes (red) materialized by circles of seven pixels in diameter. (**F**) The border of the overlaying of these circles (red spot) was defined as the *Junction*, materialized by its smoothed vectorial representation (blue). (**G**) An *Extremity* corresponds to a pixel linked to only one neighbor pixel. H: representation of one *Extremity*. See step “§10” in the Results section.

**Figure 4:**
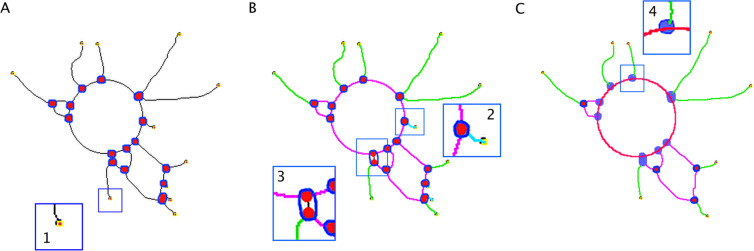
Vectorial objects detection in skeletonized trees. (**A**) Extremities, red dots surrounded in yellow (inset 1) as explained in the Methods section (§10). (**B**) detection of Branches (green) and Segments (magenta) (§11). Inset 2 shows an artifactual branch (cyan) (because too small) which will be removed by the program. Inset 3 shows a fusion between two nearby Junctions into a single one (blue edge). (**C**) representation of the final analysis, including the Anchorage Junctions (violet, inset 4), which intersect with the sphere limit (red). “§” labels correspond to steps described in the Results section.

Image analysis for FBA was performed using a program developed for the ImageJ software^19^. This plugin is an extension of the “Angiogenesis Analyzer” for ImageJ^18^ written in the macro language of ImageJ. The analysis can be divided into three main steps: (1) sphere detection (Fig. 1), (2) tree detection (Fig. 2) and (3) tree structure analysis of Junctions and Extremities (Fig. 3) as well as Segments, Branches and Anchorage Junctions (Fig. 4):

Sphere detection (Fig. 1): Sphere analysis in ideal conditions does not present particular difficulties. However, sphere clustering, dirt, uneven lighting due to the meniscus of the culture medium and the walls of the culture wells introduce difficulties, especially defaults of object closure. A series of image manipulations were necessary to enable sphere detection using simple threshold methods as follows:

§1 Light field correction (lfc) was performed using an artificial flat field obtained by applying a Gaussian convolution with a sigma value (**σ)** corresponding to the average sphere diameter. Uniform backgrounds were obtained by dividing the initial image (ima1) by the flat field with:

$${ima}1({lfc})={ima}/{{Gauss}}_{{\sigma }}({ima}1)$$

§2 Strong edges enhancement (enh) was then performed by log transforming the image and applying a variance filter with a radius (r) corresponding to the apparent thickness of the sphere edges (Fig. 1B) with:

$${Ima}1\,({enh})={{variance}}_{{r}}[{\log }({ima}1({lfc}))]$$

§3 Initial segmentation (spMask) was performed to get a coarse binary sphere mask by applying a threshold on ima1(enh) obtained using the « IsoData » method^31^, and binary objects resulting from the analysis were then submitted to a cycle of closing-opening operations and filling holes (Fig. 1C) with:

$${spMask}={fillHole}\{{Open}\{{fillHole}[{Close}({IsoData}({ima}\,1({enh})))]\}\}$$

§4 The interior of spheres was detected by a second segmentation (inMask) applied on the difference of images “spMask” and “ima1(enh)” (Fig. 1D). The result was then segmented by the “Minimum” threshold method^32^ and hole filling and opening binary operators were applied, with “r” the radius of the Maximum filter matrix (set to 2 pixels) (Fig. 1E) with:

$${inMask}={Open}\{{fillHole}[{MinimumThreshold}[{{Maximum}}_{{r}}({ima}1({lfc})-{spMask})]]\}$$

§5 Sphere edges were detected using binary object analysis of “inMask”. Some circles were fitted to the edges and their diameters were enlarged to take into account the thickness of the sphere envelope. Figure 1F shows the sphere edges using a red circle vectorial overlay.

Tree detection (Fig. 2): Tree detection consists of segmentation, avoiding shadows due to phase contrast lighting and small acellular structures, followed by skeletonization, as follows.

§6 Light field correction (lfc) was performed in the same manner as for sphere detection (§1), followed by noise removal using a band pass FFT (Fast Fourier Transform) filter (FFTbp).

$$Ima2(lfc)=FFTb{p}_{(v1;v2)}[ima2/(Gaus{s}_{{\boldsymbol{\sigma }}}(ima2))]$$

where “ima2” is the original image (Fig. 2A), “v1” is the minimum size of structures to keep and v2 is a value corresponding to the size of the shadows to be removed. “v2” has the same value than “**σ**”. These values depending of the image resolution are typically v1 = 1.5 and v2 = 400.

§7 Edge enhancement (enh) was performed to increase the signal of the small cellular extensions. A variance (r) filter was used to suppress the alternatively positive-negative aspect of objects inherent in phase contrast imaging as well as to increase high gradient areas, with “r”, the radius corresponding to the minimum size of structures to detect, set to 2 pixels). The histogram modal value was then subtracted to reduce the background (Fig. 2B), with:

$$ima2(enh)\,=[varianc{e}_{{\bf{r}}}(Ima2(lfc))\mbox{--}modal(varianc{e}_{{\bf{r}}}(Ima2(lfc)))]$$

§8 The image was then segmented using the “Mean” threshold method^33^, creating a binary mask (Fig. 2C). The mask was smoothed and made continuous using closing and dilation binary operators (Fig. 2D).

$$ima2(mask)=dilate\{close[MeanThreshold(ima2(enh))]\}$$

§9 The area within the circles was filled and the resulting binary mask skeletonized (Fig. 2E). Structures not in contact with the circles corresponding to the previously detected spheres were removed to avoid isolated pseudo-capillary objects. Circles in the skeleton were cleared to get the final tree (Fig. 2F).

Tree structure analysis: *Junctions* and *Extremities* detection (Fig. 3):

§10. Tree structure analysis started with *Junctions* and *Extremities* detection. To perform this, we defined three sub-elements: *Nodes*, which consisted in the minimum structure allowing a bifurcation in a skeletonized network, *Junctions* and *Extremities*. A pixel corresponded to a *Node* when it had at least 3 neighbors (Fig. 3 A-D). To avoid eventual further skeletonization artifacts, each pixel forming a *Node* was then replaced by a circular dot from 7 pixels of diameter (Fig. 3E). *Junctions* were formed by the group of dots associated to a bifurcation (Fig. 3F). A pixel was qualified as an *Extremity* when it had only one neighbor (Fig. 3G-H). Figure 3 summaries different configurations of *Nodes* leading to a *Junction* and an *Extremity*.

Tree structure analysis: *Segments*, *Branches* and *Anchorage Junctions* detection (Fig. 4):

§11. Result of step §10 (Fig. 4A) was submitted to the analysis of branch and segment content, according to the following definitions. *Branches* were lines, which are linked to one *Junction* and one *Extremity* (green elements, Fig. 4B) and *Segments* were lines connected to the main tree by two *Junctions* (magenta elements Fig. 4B). Touching *Junctions*, or too close *Junctions* (spaced less than 20 pixels apart) were fused into single *Junctions* to remove segments composed of less than two aligned cells (zoom inset 3, Fig. 4B). Artifactual small *Branches* were removed by an iterative pruning (zoom inset 2, Fig. 4B). Segments corresponding to *Circle* pieces intercepting circles selections were removed by a previously described method^34^. Using the same principle, the *Junctions* that intercepted *Circles* corresponding to sphere limits were defined as *Anchorage Junctions* (zoom inset 4 Fig. 4C). At this step, the automatic analysis resulted in a model of the sphere and its associated tree, consisting in a group of vectorial objects (*Circles*, *Extremities*, *Junctions*, *Branches*, *Segments* and *Anchorage Junctions*) (Table 1) which were counted and measured for statistical calculations.

**Table 1:** Vectorial objects (elements) characterized by the software analysis. The “Text” column refers to the figure and to the analytical step in the Methods section that describe each of the vectorial elements.

Vectorial element	Abbreviation	Short definition	Text
*Node*	Nd	Pixel having at least three neighbors	Fig. 3, §10
*Junction*	Jnc	Groups of nodes forming a bifurcation	Fig. 3, §10
*Extremity*	Ext	Pixel having only one neighbor	Fig. 3, §10
*Segment*	Seg	Binary line linked with two junctions	Fig. 4, §11
*Branch*	Br	Binary line linked with one junction and one extremity	Fig. 4, §11
*Anchorage Junction*	AchJ	Junction linking a branch or a segment to a sphere limit	Fig. 4, §11
*Isolated Element*	IsE	Binary line connected to two extremities	Fig. 5, §12
*Mesh*	Msh	Area enclosed by segments	Fig. 5, §13

### ETFA image analysis

**Figure 5:**
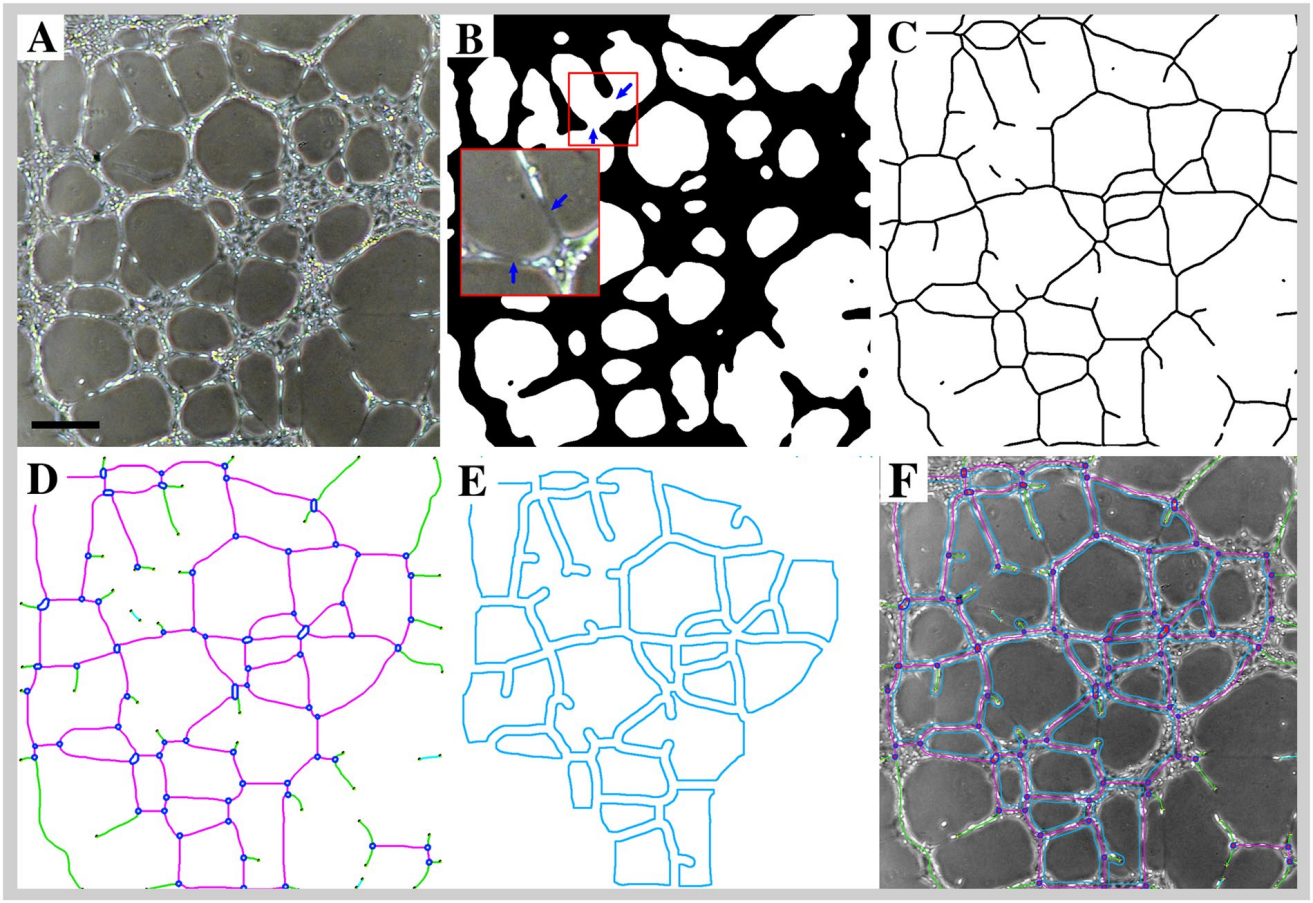
HUVEC network analysis in ETFA. (**A**) Initial image enlargement of the HUVEC network in culture. (**B**) Binary image after segmentation (§6-§8). (**C**) Skeleton of the binary segmentation (§9). (**D**) Detection of *Extremities* (dark dots), *Branches* (green), *Segments* (magenta) and *Junctions* (blue). Twigs and *Isolated Elements* that were too small to be kept in the data are in cyan. (**E**) Map of the *Meshes* consisting in closed areas surrounded by *Segments*. (**F**) Summary of the detected and vectorized objects, superimposed to the initial image. “§” labels correspond to steps described in the Results section. Scale bar: 75 µm.

§12. *Isolated Elements* detection

HUVEC cultured in Matrigel were treated to reduce noise and lighting inhomogeneity similarly than FBA images. The tree structure detection was done as in steps §6 to §9, except for the removal of the sphere area (Fig. 5A-C). Analysis of the binary skeleton was performed as explained in steps §10 and §11 except for the detection of *Anchorage Junctions*.

Elements limited by two extremities were classified as isolated elements. They were removed when the size was lower than a user defined value (10 pixels in our analysis) which corresponds to the minimum size of an isolated cell.

§13. *Meshes* detection:

HUVEC in 3D gel grow according to a meshed network. To implement a parameter allowing to quantify this level of cellular organization, a supplementary vectorial object was defined in analysis - “*Meshes*” - corresponding to closed areas delimited by *Segments* and associated *Junctions* (Fig. 5D) that were detected and measured (Fig. 5E). The final representation (Fig. 5F) summarizes all the detected structures from the initial image. Vectorial objects and measurement definitions are summarized in Tables 1 and 2, respectively.

**Table 2:** Summary of the measurement definitions performed from the vectorial objects detection.

Parameter	Abbreviation	Short definition	Text
Mean *Mesh* Size	MMS	Mean value of *Mesh* size on each image (unit: pixel)	Fig. 5 §12
Total *Mesh* Area	TMA	Sum of *Mesh* area on each image (unit: pixel)	Fig. 5 §12
Total *Segment* Length	TSL, TSL/S	Sum of *Segment* length encountered in each image (ETFA) or per *Sphere* (FBA) (unit: pixel)	Fig. 4, §11
Number of *Junctions*	JN, JN/S	Number of *Junction* per image (ETFA) or per *Sphere* (FBA)	Fig. 3, §10
Total Length	TL, TL/S	Sum of *Branches* and *Segments* length per image (ETFA) or per *Sphere* (FBA) (unit: pixel)	Fig. 4, §11
Number of *Anchorage Junction*	AJN/S	Number of *Anchorage Junction* per *Sphere*	Fig. 4, §11

**Figure 6:**
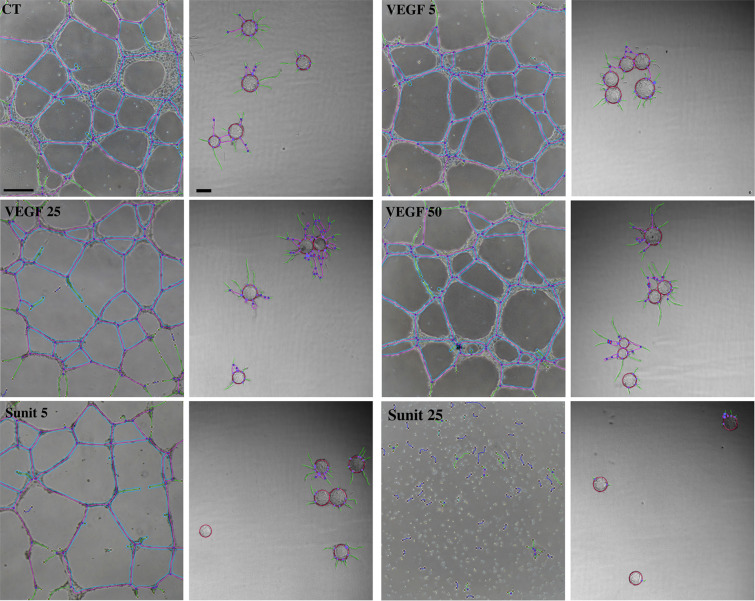
Comparative network patterns of HUVEC. ETFA (left column images) and FBA, (right column images). HUVEC were grown for 1 day (ETFA) or for 4 days (FBA) in the absence (CT, control) or presence of VEGF-A (5, 25 and 50 ng/ml) or of sunitinib (Sunit: 5 and 25 nM). Phase contrast images with the superposition of vectorial objects obtained from computer analysis using the customized “Angiogenesis Analyzer” for ImageJ are shown: green, *Branches*; magenta, *Segments*; red surrounded by blue, *Junctions*; cyan, *Meshes*; violet, *Anchorage Junctions*; red circle, *Sphere*. ETFA scale bar, left: 100 µm. FBA scale bar, right: 200 µm.

**Figure 7:**
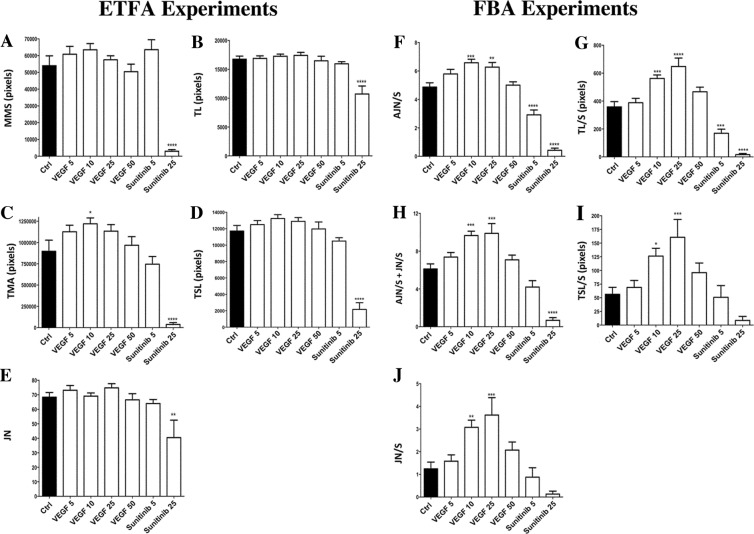
Comparative measurement of parameters obtained from image analysis in ETFA and FBA. ETFA: HUVEC were cultured for 1 day in Matrigel; FBA: HUVEC were cultured for 4 days on the surface of Cytodex 3 microbeads. Crtl, medium only (black bars); increasing concentrations of VEGF-A (5, 10, 25 and 50 ng/ml) and sunitinib (5 and 25 nM) (white bars). (**A**) MMS: Mean *Mesh* Size; (**B**) TL: Total Length; (**C**) TMA: Total *Mesh* Area; (**D**) TSL: Total *Segment* Length; (**E**) JN: Number of *Junction*; (**F**) AJN/S: Number of *Anchorage Junction* per sphere; (**G**) TL/S: sum of *branches* and *segments* length per *Sphere*; (**H**) AJN/S + JN/S: sum of number of *Anchorage Junction* and *Junction* per sphere; (**I**) TSL/S: sum of *segment* length per *Sphere;* (**J**) JN/S: Number of *Junction* per sphere. Common parameters are Number of *Junction* (JN), Total Length (TL) and Total *Segment* Length (TSL). Statistical analyses were performed by One-way ANOVA followed by Dunett’s multiple comparison test. Each result in the presence of VEGF-A or Sunitinib was compared to the corresponding Ctrl experiment. (*p < 0.05; **p < 0.01; ***p < 0.001; ****p < 0.0001). Error bars correspond to the number of analysed images (n = 15 acquired from 3 wells of 190 mm^2^ for ETFA, and n = 32 acquired from 8 wells of 32 mm^2^ for FBA) ± SEM providing from one experiment performed with each method used.

We further decided to test and compare this two algorithms on the reliability, accuracy and statistical relevance of some measurements obtained on two sets of FBA and ETFA experiments. These assays were performed in parallel on HUVEC cells, using both activating (VEGF-A) and inhibiting (sunitinib) conditions of angiogenesis. Images from cellular organization were acquired from ETFA and FBA through phase contrast microscopy (Fig. 6) and analyzed with the corresponding algorithms to detect the different vectorial objects described above (Table 1). We finally made a comparative analysis of the parameters derived from the object measurements (Table 2), (Fig. 7).

### The endothelial tube formation assay response

HUVEC in culture wells present a wide range of aspects after one day of culture, as observed by phase contrast microscopy, from short and isolated segments to a highly developed meshing, depending on the treatment conditions (Fig. 6). Quantitative analysis of different angiogenic parameters derived from AA on EFTA are summarized in Fig. 7, panel A to E.

**Figure 8:**
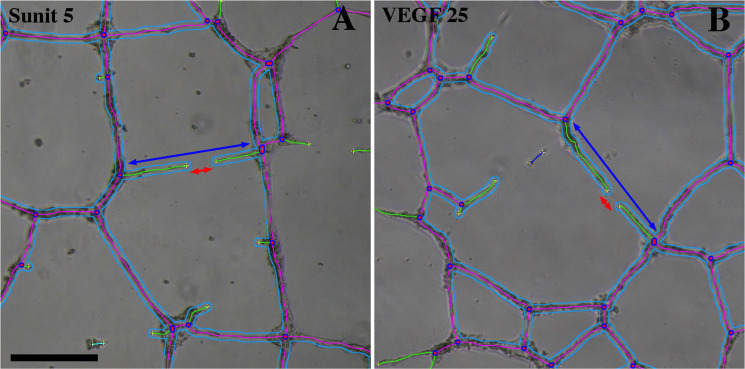
*Segment* disruption and *Mesh* extension in ETFA. Image enlargement of HUVEC that have been cultured for 24 h in Matrigel in inhibitory (sunitinib, 5 nM), (**A**) or stimulatory (VEGF-A, 25 ng/ml) conditions (**B**). A red double-head arrow was set in each picture as an example of *Segment* disruption leading to regression of a capillary-like motive and consequently to *Mesh* extension. Lengths of the red arrows correspond to loss of size in the initial *Segment* of which only two green branches remain. This size decrease is very modest when considering “Total Length” value (magenta *Segments* + green *Branches*). Blue double-head arrows show the loss of size - much higher - in “Total Segment Length” which does not take into account the branches contribution to the global tree structure. Scale bar: 100 µm.

Compared to control condition, the mean size of HUVEC meshes (MMS) slightly increased when VEGF-A concentrations went from 5 to 10 ng/ml, and then decreased with higher concentrations (25 and 50 ng/ml) (Fig. 7A). Although this effect is not statistically significant (VEGF-A 10/Ctrl = 1.18, NS), the tendency is still clearly visible. The same biphasic effect of VEGF-A increasing dose was significantly observed when considering the Total Mesh Area (TMA) (VEGF-A 10/Ctrl = 1.36 (p < 0.05)) (Fig. 7C). While this tendency was still visible with Total Segment Length (TSL) (VEGF-A 10/Ctrl = 1.13 NS) (Fig. 7D), it became erratic in terms of Junction Number (JN) and Total Length (TL) values (Fig. 7B,E). This can be explained by considering the kinetic of the meshing network structuration during HUVEC culture in Matrigel. As previously observed in time-lapse recording of EC in similar culture conditions (video at this link: http://image.bio.methods.free.fr/ImageJ/?Human-Endothelial-Progenitor-Cells-in-vitro-tube-forming-analysis-using-Lens), the network establishment started (3–6 h of culture) by the formation of a multitude of small and unstable meshes. The size of the meshes then progressively increased by fusion of proximal meshes, which occurred by segments (tube) regression or disruption. Indeed, disruption of a segment can lead to the transformation of two adjacent meshes in a single one, as regression of a segment can lead to regression of a mesh until its disappearance. Examples of this phenomenon are shown in Fig. 8. Red double-head arrows show segment disruption leading to meshes fusion by opening a breach in segment continuities. Residual branches maintained the constant Junction Number (JN) at this early stage of the regression and the decrease of the Total Length (TL) was negligible. Although the disruption leads to a higher decrease in Total Segment Length (TSL) by a length value that corresponds to the double head blue arrow (Fig. 8B), such phenomena is not sufficient to be significant in the TSL measurements in the VEGF-A response (Fig. 7D). MMS increased as TMA, segment disruptions and mesh fusion leading to an increase in residual meshes (Fig. 7A,C). This tendency reversed when mesh size became too high to be entirely visible in the image field or when meshes became broken. At this step (VEGF-A 25 and 50, Fig. 7A,C), contribution of big meshes decreased, as the mean values of MMS and TMA.

In the presence of 5 nM sunitinib, the capillary-like network was not significantly increased (MMS Sunit 5/Ctrl = 1.18 (NS)). Comparatively, the network was highly altered with 25 nM sunitinib (MMS Sunit 25/Ctrl = 0.06 (p < 0.0001)) (Fig. 6,8A). This apparent contradiction - there was a non significant increase of MMS with 5 nm sunitinib (Fig. 7), while a decrease would have been expected - can be explained by the process of mesh growing. As indicated above, the increase in mesh size occurs by segment disruption, thus leading to mesh fusion. A slight inhibitory effect affecting the tube organization as a function of time has the same effect - segment disruption and mesh fusion (Fig. 8A). This makes the use of MMS value inappropriate in the presence of a low concentration of inhibitor. Interestingly, the TMA and TSL reflected a slight decrease with 5 nM sunitinib (TMA Ctrl/Sunitinib = 1.20 NS) (Fig. 7C,D). This is consistent since mesh fusion did not increase the TMA whereas it decreased the TSL (Fig. 8). When segment disruptions occured in large scale, a strong and significant inhibition was observed on TMA values (Ctrl/sunitinib 25 = 24 (p < 0.0001)) (Fig. 7C). The high level of inhibition (sunitinib 25 nM) led to a dramatic alteration of the network, as reflected by significant differences in other parameters: JN Ctrl/sunitinib 25 = 1.7 (p < 0.01) and TSL Ctrl/sunitinib 25 = 5.4 (p < 0.0001).

### The fibrin bead assay response

In the FBA model, each microbead consists in a little local experiment in terms of EC loading and spatial HUVEC tube growing, and can be considered as an assay by itself. Pseudo-capillary growth on micro beads presents variability, smoothed by the number of analyzed elements (about 30 beads analyzable per well, 180 for each dose in this study). Depending on treatment conditions, images revealed diverse structural aspects going from a few number of small branches anchored to a sphere (Fig. 6 Sunit 25) up to developed trees composed of several segments and branches (Fig. 6, VEGF-A 25). All parameters exhibited a biphasic response to VEGF-A and a dose-dependent inhibition with sunitinib (Fig. 7). Within the best responses in terms of treated/control ratio, Junction Number/Sphere (JN/S), Total Length/Sphere (TL/S) and Total Segment Length/Sphere (TSL/S) VEGF-A/Ctrl ratios were respectively 2.9 (p < 0.001), 1.8 (p < 0.0001) and 2.9 (p < 0.001) for the maximal activation at 25 ng/ml (Fig. 7G,I,J). Among these three measurements, TSL/S and JN/S gave the best results in terms of response amplitude to the treatment, under strong or low activator concentrations. This shows the superiority of using segment and junction detection instead of the basic total length of capillaries. The Number of Anchorage Junction/Sphere (AJN/S), which are specific measurements of FBA and can be associated to the capacity to initiate sprouting, gave the smallest SEM for the maximum activation at the dose VEGF-A 10 compared to control conditions (AJN/S VEGF-A 10/Ctrl = 1.35 (p < 0.001)). Another measurement associating a precocious phenomenon (initiating of sprouting) with a later one revealing the tree complexity, consists in the sum of AJN/S and JN/S (Fig. 7H). Values from these measurements gave a nearly similar inhibition/activation profile than AJN/S only, with a higher control ratio (1.6 (p < 0.001)), although with higher SEM. It is interesting to note that the maximum response to VEGF-A occurred at the dose of 10 ng/ml considering AJN/S (as for TMA in the ETFA assay) instead of 25 ng/ml for other measurements. The FBA assay gave consistent results with the sunitinib inhibitor, with significant inhibitions (p < 0.0001) for the higher dose of 25 nM using the three following measures: AJN/S, TL/S and AJN/S + JN/S (Fig. 7F,G,H), as expected. The dose of 5 nM showed also significant differences compared to the control condition with the TL/S and the AJN/S measurements with a control ratio of 2.1 (p < 0.001) and 1.7 (p < 0.0001), respectively.

### Comparison of ETFA and FBA responses

The ETFA and FBA tests are not directly statistically comparable, since respective size of culture wells involved a different number of replicates: 3 wells for ETFA (190 mm^2^, total of 570 mm^2^ cultured), and 8 for FBA (32 mm^2^, total of 256 mm^2^ cultured). Even with a higher cultured area for ETFA compared to FBA (about 2-fold more), a significant difference compared to the control was only found with one VEGF-A treatment (VEGF-A 10 ng/ml, p < 0.05), and with TMA parameter only using ETFA. In the case of FBA, several VEGF-A doses showed significant differences (p < 0.01 or lower), considering all parameters (Fig. 7). As mentioned above, each sphere can be assimilated to a single test in itself, and thus, the high number of analyzed spheres (about 30 per well) resulted in high reproducibility, which is an advantage for this method.

Another difference between the ETFA and FBA methods was the amplitude of the response amongst the various treatment doses in each method. The strongest response was found with a dose of 10 ng/ml VEGF-A in ETFA experiments, with a ratio VEGF-A 10/Ctrl = 1.36 (p < 0.05) when measuring TMA (Fig. 7C). The same condition using the FBA method showed ratios going from 1.35 (for AJN/S) to 2.46 (for JN/S), and 2.24, 1.57 and 1.57 for TSL/S, TL/S and AJN + JN/S, respectively. Except for the *Anchorage Number* (AJN/S), other parameters exhibited a higher treatment/control ratio. This can be explained as the early meshing structure of HUVEC constrained differences in amplitude in ETFA. In the case of FBA, pseudo-capillary growth from the sphere surface is not spatially limited and therefore differences in measurements were bigger than with ETFA.

It is interesting to note that VEGF-A 10 induced nearly the same maximum response regarding AJN/S (ratio over control 1.35) in the FBA assay than for TMA (ratio over control 1.36) in the ETFA assay. While AJN/S can be assimilated to a measurement of sprouting of EC, a precocious phenomenon, other FBA parameters (TL/S, TSL/S and JN/S) reflect later development processes, like segment elongation and/or pseudo-vascular bifurcations. They exhibit a maximum response for the VEGF-A dose of 25 ng/ml.

When measuring inhibitory activities, FBA proved to be more powerful and sensitive than ETFA to measure small changes or weak inhibitors.

## Discussion

In this comparative analysis between ETFA and FBA methods, we extended the “Angiogenesis Analyzer” to enable FBA analysis, including sphere detection and pseudo-vessel organization. The extended “Angiogenesis Analyzer” is written in the macro language of ImageJ, free image analysis software from the National Institutes of Health^19^. While other software offers some solutions for analysis of images acquired by fluorescence complex confocal imaging^35^, here we focused on the analysis of images issued from phase contrast high-throughput microscopy. Although three-dimensionally distributed, the extensions of micro-capillaries preferentially developed in the horizontal plane. Thanks to a short, but sufficient depth of field, microscopy observation in phase contrast was found convenient enough to get a good sampling of field deepness containing pseudo-capillary developments (not shown). Furthermore, although more difficult to analyze, phase contrast observation method does not require any previous treatment of cells, has no phototoxicity and can therefore be used for kinetic experimentations and time-lapse recording.

Although widely used for ETFA analysis (more than 150 citations), the “Angiogenesis Analyzer” has never been described in details. Here, we report the algorithm and describe the meaning of the detected and modelled structures. This may facilitate the analysis of ETFA when following published protocols, which advise the use of “Angiogenesis Analyzer”^36^. This method has the advantage of being easy to implement. In this work, we bring out some limitation in terms of sensitivity, especially for angiogenesis inhibitors with low activities that cannot be identified.

Similarly, we demonstrate here that the “FBA algorithm” is adapted for performing precise analyses of pseudo-capillary growing on microbeads. We overrode the constraint of using phase contrast and also implemented the method in 96 well plates with automatic image acquisition. By using this new approach, we dramatically increased the repeatability and precision of angiogenesis measurements.

We observed a biphasic profile of VEGF-A treatment with the two methods, ETFA and FBA. This biphasic effect of VEGF-A has already been reported in similar *in vitro* angiogenesis assays^37,38^, and can be explained by a dose-dependent effect of the growth factor on the expression of its cognate receptors^39^, or by the effect of accessory molecules interfering with VEGF-A signaling^40^. While this similar profile can be observed with both methods, the amplitude of difference between control conditions and VEGF-A treatment is far more precise with the FBA method, and moreover by using a smaller total cell culture area. In ETFA, the measured parameters are mainly followed by the regression or growing of the meshing, which is spatially limited by the network itself. This limits the amplitude of the measured difference between a control condition and an effector. In the case of FBA, the growth in the first 3 days of culture is not spatially limited. This and the high number of analyzed spheres, each of them being considered as an assay in itself, yields accuracy and repeatability superiority to that obtained with ETFA. Remarkably the maximum of biphasic VEGF curves was the same in both cases (10 ng/mL ETFA-TMA and FBA-AJN/S), although the two sets of experiments were performed in two different laboratories, with two different HUVEC and VEGF sources.

An advantage of the FBA method is that it is easily adapted to small culture wells. Indeed, the meniscus of the gel medium imposes a relatively large area of culture to get a reasonable representative EC network sample into single image acquisition on ETFA. On FBA, the small size of beads and their associated endothelial cellular extensions, allow the encompassment of this limitation. Another advantage of the FBA, is to allow the analysis of several aspects of angiogenesis: sprouting (*Anchorage Junction* detection), as well as elongation and bifurcations, which are measured through parameters qualifying and quantifying segments, junctions and branches organization of pseudo-capillaries. Moreover, the use of tightly packed cells on carrier microbeads represents characteristic features of an *in vivo* environment in this assay. Indeed, ETFA appears more limited to an early mix of sprouting and growing capacities occurring at the precocious meshing establishment. However, both assays can be run in a relatively high throughput manner, allowing vascular studies at a genetic, molecular, or pharmacological level.

In conclusion, we demonstrated that phase contrast images of ETFA and FBA experiments could be successfully analyzed by the customized ImageJ’s “Angiogenesis Analyzer”, from small image samples, to large batches of images. We also shed light on the advantages and disadvantages of both methods; ETFA in 24 wells plates being easy to implement, although less precise than FBA in 96 well plates, the latter approach requiring higher technicality. ETFA on the other side is faster to perform, with only 24 h of cell culture instead of 4 days for FBA. The present comparison of the ETFA and FBA methods demonstrates their robustness. More generally, our approach in terms of image analysis represents a valuable and robust tool to the study of basic branching systems, related to the angiogenesis process. It can also be used as alternative applications in the analysis of neural network or of any other biological or physical systems in which the accurate measurement of branching or meshing morphology complexity is of interest. As we observed that segments, junctions and branches provide a good representation of low developed networks and trees, we also recognize that more complex microvascular architectures in highly developed biological structures may require more elaborate descriptors. Finally, using similar approaches, it may be possible to upgrade our present version of “Angiogenesis Analyzer” in order to analyze *in vivo* microvascular structures to better characterize tumor angiogenesis.

## Methods

### Reagents

FBA: HUVEC and NHDF, Endothelial basal medium-2 (EBM-2), complete fibroblast growth medium (complete FGM) culture media, Supplement Mix, human fibroblast growth factor-2 (FGF2), long R insulin-like growth factor-1 (R3-IGF-1)), human epidermal growth factor (EGF) were from PromoCell (Heidelberg, Germany), fetal bovine serum (FBS) from Gibco. Ascorbic acid, hydrocortisone and heparin were from Sigma Aldrich, penicillin and streptomycin from Invitrogen, Cytodex 3 microcarriers beads from GE Healthcare Europe GmbH (Freiburg, Germany). Sunitinib and human vascular endothelial cell growth factor-A (VEGF-A) were from R&D Systems (Minneapolis, USA). All reagents were of culture grade.

ETFA: HUVEC cells were purchased from ABCell-Bio (Evry, France), culture medium endothelial growth medium 2-microvascular (EGM2-MV) was from Lonza (Basel, Switzerland), Matrigel from BD (Le Pont de Claix, France) and VEGF-A was from Miltenyi (Paris, France). All reagents were of culture grade.

### Cell culture

FBA: HUVEC were cultured in EBM-2 with 1% penicillin/streptomycin supplemented with 2% Supplement Mix, thus constituting the EGM-2 as previously described^14^. In all experiments, cells between passages 2 and 7 were used. NHDF from juvenile foreskin were cultured in complete FGM supplemented with 5 µg/mL insulin and 1 ng/mL hFGF2 (human FGF2). NHDF were used between passages 2 and 9. All cells were cultured in 5% CO_2_ at 37 °C and media were replaced every 2 days.

ETFA: HUVEC were seeded at 5 000 cells/cm^2^ and cultured in EGM-2 MV medium. Media was changed every 2 days. Assays were performed between passages 5 and 10.

### Endothelial tube formation assay

Network formation in the ETFA was carried out by seeding HUVEC (10^5^ cells/well) on Matrigel (250 µl/well) into a 24-well plate for 24 h at 37 °C with 5% CO_2_. Cells were suspended in EGM2-MV medium without VEGF-A (control condition), or complemented with 5, 10, 25 or 50 ng/ml VEGF-A. Sunitinib, a multitargeted tyrosine kinase inhibitor was added (5 nM or 25 nM) to culture medium as an angiogenesis inhibitor. Five pictures per well (center of the well and four cardinal points) were taken at time 24 h using a camera Nikon D5300 associated to an inverted microscope Nikon Eclipse TS100 using a 4× objective (NA 0.13) in phase contrast mode without fixation. For statistical analyses, three wells were seeded per conditions.

### Fibrin bead assay

FBA was carried out using HUVEC on dextran-coated Cytodex 3 microcarriers beads embedded in fibrin gel as previously described^14^. Dry Cytodex 3 microcarrier beads were hydrated in PBS for at least 3 h at RT and autoclaved for 15 min at 115 °C. The culture medium used in the assay was EGM-2. EGM-2 corresponded to EBM-2 medium supplemented with 2% [v/v] FBS, 10 ng /ml FGF2, 5 ng/ml EGF, 0.5 ng/ml VEGF-A, 20 ng/ml R3-IGF-1, 1 µg/ml ascorbic acid, 0.2 µg/ml hydrocortisone and 22.5 µg/ml heparin. HUVEC were mixed with Cytodex 3 microcarrier beads at a cell density of 400 cells per bead in a solution of 2500 beads per ml of EGM-2 medium. Beads and HUVEC were then co-incubated in a humidified incubator at 37 °C and 5% CO_2_ and gently manually shaken every 20 min for 4 h to allow cell adherence to the bead surface. Beads with adherent cells were transferred to a 75 cm^2^ tissue culture flask and were further incubated for 24 h. Cells coated on beads were then washed three times with 1 ml of EGM-2 to remove non-coated cells and were resuspended at a density of 1000 beads/ml in a solution of fibrinogen type I (2.5 mg/ml) with 0.15 U/ml of aprotinin at a pH of 7.4. In order to polymerize the fibrin in the wells, four µl of 10 units/ml of thrombin were deposited into each well of a 96-well-optical plate (BD Falcon) suitable for high throughput imagers. Then, 80 µl of the fibrinogen type I-aprotinin-HUVEC coated bead solution were delivered over the thrombin drops, gently mixed by slowly pipetting up and down and allowed to clot for 2 min at room temperature and then at 37 °C and 5% CO_2_ for 30 min to promote gel formation. Eighty µl of EGM-2 medium were added to each well and allowed to equilibrate with the bead-containing gels for 30 min at 37 °C and 5% CO_2_. Afterwards, 3700 NHDF were added and allowed to adhere to the top of the gel. After 1 h, EGM-2 was replaced with fresh medium with or without VEGF-A or sunitinib. Medium was changed one day after and then every two days. Sprouting was apparent between days 2 and 3 and cultures were imaged at day 4. In order to quantify the sprouting pseudo-microvessel network, samples were automatically scanned with a high-throughput imager (IXM, Molecular Device) at a 4x magnification with definition of 4 quadrants (images) per well to cover the entire surface of each well. Eight wells were analyzed per condition. The image analysis described further was performed on the largest squared area contained in the lightening area of quadrant images.

### Image analysis

Image analysis for the FBA and ETFA were performed using a program developed for the ImageJ software^19^. This plugin is an extension of the “Angiogenesis Analyzer” for ImageJ^18^ written in the macro language of ImageJ, which is described in the Results section.

### Statistical analysis

Data are expressed as means ± SEM as previously decribed^14^. One-way ANOVA was used for multiple comparisons in experiments with one independent variable. A Dunnet’s test was used for post hoc analysis of the significant ANOVA. A difference in mean values between groups was considered to be significant when p ≤ 0.05. Error bars correspond to the number of analysed images (n = 15 for ETFA, and n = 32 for FBA) ± SEM providing from one experiment performed with each method used.

## References

[1] Carmeliet P (2003). Angiogenesis in health and disease. Nat. Med..

[2] Bergers G, Benjamin LE (2003). Tumorigenesis and the angiogenic switch. Nat. Rev. Cancer.

[3] Logsdon EA, Finley SD, Popel AS, Mac Gabhann F (2014). A systems biology view of blood vessel growth and remodelling. J. Cell Mol. Med..

[4] Kerbel R, Folkman J (2002). Clinical translation of angiogenesis inhibitors. Nat. Rev. Cancer.

[5] Ferguson FM, Gray NS (2018). Kinase inhibitors: the road ahead. Nat. Rev. Drug. Discov..

[6] Ferratge S (2017). Initial clonogenic potential of human endothelial progenitor cells is predictive of their further properties and establishes a functional hierarchy related to immaturity. Stem Cell Res..

[7] Staton CA, Reed MW, Brown NJ (2009). A critical analysis of current *in vitro* and *in vivo* angiogenesis assays. Int. J. Exp. Pathol..

[8] Madu C, Li L, Lu Y (2016). Selection, Analysis and Improvement of Anti-Angiogenesis Compounds Identified by an Anti-HIF-1alpha Screening and Validation System. J. Cancer.

[9] Montesano R, Orci L, Vassalli P (1983). *In vitro* rapid organization of endothelial cells into capillary-like networks is promoted by collagen matrices. J. Cell Biol..

[10] Montesano R, Orci L (1985). Tumor-promoting phorbol esters induce angiogenesis *in vitro*. Cell.

[11] Goodwin AM (2007). *In vitro* assays of angiogenesis for assessment of angiogenic and anti-angiogenic agents. Microvasc. Res..

[12] Nowak-Sliwinska P (2018). Consensus guidelines for the use and interpretation of angiogenesis assays. Angiogenesis.

[13] Nehls V, Drenckhahn D (1995). A novel, microcarrier-based *in vitro* assay for rapid and reliable quantification of three-dimensional cell migration and angiogenesis. Microvasc. Res..

[14] Berndt S, Issa ME, Carpentier G, Cuendet M (2018). A Bivalent Role of Genistein in Sprouting Angiogenesis. Planta Med..

[15] Issa ME, Berndt S, Carpentier G, Pezzuto JM, Cuendet M (2016). Bruceantin inhibits multiple myeloma cancer stem cell proliferation. Cancer Biol. Ther..

[16] Nakatsu MN, Hughes CC (2008). An optimized three-dimensional *in vitro* model for the analysis of angiogenesis. Methods Enzymol..

[17] Queiroz MMF (2018). NF-kappaB and Angiogenesis Inhibitors from the Aerial Parts of Chresta martii. J. Nat. Prod..

[18] Carpentier, G. Angiogenesis Analyzer for ImageJ. *ImageJ News* **9 November****2012** (2012).

[19] Rasband, W. S. *ImageJ. U. S. National Institutes of Health*, Bethesda, Maryland, USA (1997-2020).

[20] Marona P (2017). MCPIP1 Downregulation in Clear Cell Renal Cell Carcinoma Promotes Vascularization and Metastatic Progression. Cancer Res..

[21] Sultani AB, Marquez-Curtis LA, Elliott JA, McGann LE (2016). Improved Cryopreservation of Human Umbilical Vein Endothelial Cells: A Systematic Approach. Sci. Rep..

[22] Fortenberry YM, Brandal SM, Carpentier G, Hemani M, Pathak AP (2016). Intracellular Expression of PAI-1 Specific Aptamers Alters Breast Cancer Cell Migration, Invasion and Angiogenesis. PLoS One.

[23] Samarelli AV (2014). Neuroligin 1 induces blood vessel maturation by cooperating with the alpha6 integrin. J. Biol. Chem..

[24] Cossutta, M. *et al*. Weibel-Palade Bodies Orchestrate Pericytes During Angiogenesis. *Arterioscler Thromb Vasc Biol*, Atvbaha119313021, 10.1161/atvbaha.119.313021 (2019). 10.1161/ATVBAHA.119.313021 31315435

[25] Javaheri N (2015). Temperature affects the silicate morphology in a diatom. Sci. Rep..

[26] Soltani A (2017). Increased signaling by the autism-related Engrailed-2 protein enhances dendritic branching and spine density, alters synaptic structural matching, and exaggerates protein synthesis. PLoS One.

[27] Khoo CP, Micklem K, Watt SM (2011). A comparison of methods for quantifying angiogenesis in the Matrigel assay *in vitro*. Tissue Eng. Part. C. Methods.

[28] Boizeau ML (2013). Automated image analysis of *in vitro* angiogenesis assay. J. Lab. Autom..

[29] Chevalier F (2014). Glycosaminoglycan mimetic improves enrichment and cell functions of human endothelial progenitor cell colonies. Stem Cell Res..

[30] Sakr OS (2016). Arming embolic beads with anti-VEGF antibodies and controlling their release using LbL technology. J. Control. Rel..

[31] Ridler TW, Calvard S (1978). Picture thresholding using an iterative selection method. IEEE Trans. Systems, Man. Cybern..

[32] Prewitt JM, Mendelsohn ML (1966). The analysis of cell images. Ann. N. Y. Acad. Sci..

[33] Glasbey CA (1993). An analysis of histogram-based thresholding algorithms. CVGIP: Graph. Model. Image Process..

[34] Carpentier, G. Sort Selections in Overlays. *ImageJ News* **2013** (2013).

[35] Eglinger J, Karsjens H, Lammert E (2017). Quantitative assessment of angiogenesis and pericyte coverage in human cell-derived vascular sprouts. Inflamm. Regen..

[36] DeCicco-Skinner, K. L. *et al*. Endothelial cell tube formation assay for the *in vitro* study of angiogenesis. *J Vis Exp*, e51312, 10.3791/51312 (2014). 10.3791/51312 PMC4540586 25225985

[37] Chen Z (2009). *In vitro* angiogenesis by human umbilical vein endothelial cells (HUVEC) induced by three-dimensional co-culture with glioblastoma cells. J. Neurooncol.

[38] Nakatsu MN (2003). VEGF(121) and VEGF(165) regulate blood vessel diameter through vascular endothelial growth factor receptor 2 in an *in vitro* angiogenesis model. Lab. Invest..

[39] Meng H (2006). Biphasic effects of exogenous VEGF on VEGF expression of adult neural progenitors. Neurosci. Lett..

[40] Shin WS, Na HW, Lee ST (2015). Biphasic effect of PTK7 on KDR activity in endothelial cells and angiogenesis. Biochim. Biophys. Acta.

